# Awareness of Contraceptives and Their Use Among Saudi Women Attending Primary Care Centers in King Abdulaziz Medical City, Riyadh, Saudi Arabia

**DOI:** 10.7759/cureus.48820

**Published:** 2023-11-14

**Authors:** Ramah Ashoor, Sarah Alrashid, Sahar Alruhaimi, Shuq Alanazi, Hadeel Alzahrani, Yara S Alshammari, Alhanoof Alotaibi

**Affiliations:** 1 Medicine, King Saud Bin Abdulaziz University for Health Sciences College of Medicine, Riyadh, SAU; 2 Medicine and Surgery, King Saud Bin Abdulaziz University for Health Sciences College of Medicine, Riyadh, SAU; 3 Ophthalmology, King Saud Bin Abdulaziz University for Health Sciences College of Medicine, Riyadh, SAU; 4 Ophthalmology, King Abdulaziz Medical City, Riyadh, SAU; 5 Family Medicine, King Abdulaziz Medical City, Riyadh, SAU

**Keywords:** contraception, knowledge, types of contraceptives, awareness, contraceptive use prevalence, primary care

## Abstract

Background

The objective of this cross-sectional study is to identify the prevalence of contraceptive use and the knowledge and attitudes of Saudi women towards it.

Methods

We distributed a survey to Saudi women aged 19-49 attending primary care centers under King Abdulaziz Medical City in Riyadh to identify their views on using contraceptives and what they know about them. We calculated the sample size using the Roasoft sample calculator.

Results

This study enrolled 432 Saudi women. The number of women who were contraceptive users was 249 (57.6%). Among those who were using contraceptives, the most common reason was the idea of taking care of themselves and avoiding consecutive pregnancies (105, 42.2%). Of the non-users, the most common reason was concerns regarding side effects (41%). The most commonly used contraceptive methods were contraceptive pills (55.6%) and intrauterine devices (IUDs) (17.6%). The most commonly used non-pharmacological contraception methods were withdrawal (17.6%) and rhythm (8.6%).

Conclusion

In this study, factors associated with contraceptive use among Saudi women were explored. Demographic data, type, attitude, and associations provided insight into factors taken into consideration while developing future contraceptives in addition to improving clinical practice.

## Introduction

The use of contraceptives is one of the greatest advancements in modern medicine. It permits women of reproductive age to prevent unwanted pregnancies, reach their desired number of children, and plan their desired interval between children [1]. The use of contraceptives in developing countries has been correlated with reduced maternal mortality by 40%, and proper child spacing has been linked to better perinatal outcomes [2]. Contraceptive use offers a range of non-health-related outcomes, such as economic advancement for countries and sustainable population growth, as studies have shown that the total fertility rate of a nation is inversely related to the prevalence rate of contraceptive use [2, 3].

Women in the Middle East have encountered rapid changes in the past few decades, with more women entering the workplace and acquiring higher education [4]. The rapid change in the socio-demographic pattern in Saudi Arabia, especially, will result in changed behaviors and attitudes towards fertility, with more couples adopting contraceptives and different family planning methods [5].

In Saudi Arabia, the use of contraception is highly affected by several factors, such as the number of children, educational level, working conditions, parents age, the gender of the last child in the family, and other cultural, psychological, and methodical factors [5-7]. Oral and intrauterine contraceptives were the most commonly used methods of contraception [8-10]. A recent survey conducted in 2018 by the Saudi Household Health Survey, and concluded that the overall prevalence of contraception use was 30.4% in 2018 [11]. However, it's considered to be relatively low compared with other underdeveloped countries [12, 13]. Since Saudi Arabia isn't widely using family planning, the 2030 vision is highly directed to play a major role by supporting family planning programs and empowering Saudi women all over the country to eliminate all the obstacles that keep them from using different methods of contraception [11, 14]. In this study, our focus is to answer these questions: What do women know about contraceptives? What are their ideas about using contraceptives? Is there any relation between using contraceptives, contraceptive knowledge, and women’s socio-economic class?

## Materials and methods

Study design, area, and setting

This study was cross-sectional and was conducted using a self-administered questionnaire. It took place in primary care centers under King Abdulaziz Medical City (KAMC) in Riyadh, Saudi Arabia. The survey was distributed among attendees at the governmental primary care centers at KAMC.

Study subjects, sample size, and sampling techniques

The study included Saudi female attendees of KAMC primary health centers who had ever been married, with or without children, and aged 18-49 years. We excluded females under the age of 18, non-Saudi women, menopausal women, and females who had never been married. The sample size was calculated via the Raosoft sample calculator (Raosoft Inc., Seattle, WA) to assess a sample size of 20,000 with a margin of error of 5% and a confidence level of 75%, yielding a recommended sample size of 377. The sampling technique used was non-probability convenience sampling, and before data collection, the agreement and consent of participants were obtained.

Data collection process

The data were collected in different KAMC centers in Riyadh during working hours. After getting consent and permission from the participants, the research members and data collectors instructed them to scan the barcode or fill out the questionnaire on the designated tablets. The questionnaire was originally developed by Dr. Mounira Al Sheeha [6]. It is a valid, reliable questionnaire that has taken the participants about 10 minutes to complete. It consisted of three sections (socio-demographics of the participants, attitudes, and use of contraception).

Data analysis and statistical analysis

Data were retrieved from the questionnaire, arranged into a Google sheet (Google LLC, Mountainview, CA), and saved and secured by research members. It was then reviewed and cleaned for analysis. The data were presented as numbers and percentages for all categorical variables. The relationship between the use of contraceptives and socio-demographic characteristics has been performed using the Chi-square test. Based on the significant results, a multivariate regression analysis was subsequently performed to determine the significant independent predictor associated with the use of contraception, with a corresponding odds ratio and a 95% confidence interval. A p-value of less than 0.05 was taken as statistically significant. All data analyses were performed using IBM SPSS software version 26 (IBM Corp., Armonk, NY).

## Results

This study enrolled 432 Saudi women. In the study group, 71.5% were aged 30 years or older. Nearly 60% were university degree holders, and 45.4% had monthly incomes of 5,000 to 10,000 SAR (Saudi Riyal). Approximately two-thirds (65%) were unemployed, and 45.1% had a parity of one to three. Acceptance of contraceptive use was reported by 84.5% of the women, while husbands' acceptance was reported by 80.8%. In addition, 56.7% desired to have four to six children with a birth interval of two to three years (50.5%) (Table 1).

**Table 1:** Sociodemographic characteristics of the Saudi women included in the study (n=432)

Study variables	N (%)
Age group	
<30 years	123 (28.5)
≥30 years	309 (71.5)
Educational level	
Uneducated	39 (09.0)
Less than high school	46 (10.6)
High school	93 (21.5)
University education	254 (58.8)
Family monthly income (SAR)	
<5,000	108 (25.0)
5,000 - 10,000	196 (45.4)
10,001 - 15,000	30 (06.9)
15,001 - 20,000	61 (14.1)
20,001 - 25,000	19 (04.4)
>25,000	18 (04.2)
Occupational status	
Unemployed	281 (65.0)
Employed	145 (33.6)
Free business	06 (01.4)
Parity	
None	53 (12.3)
1-3	195 (45.1)
4-6	121 (28.0)
7 or more	63 (14.6)
Acceptance of the use of contraceptives	
Yes	365 (84.5)
No	67 (15.5)
Husband's acceptance of the use of contraceptives	
Yes	349 (80.8)
No	83 (19.2)
Desired number of children	
None	06 (01.4)
1	11 (02.5)
2-3	103 (23.8)
4-6	245 (56.7)
7-10	46 (10.6)
More than 10	21 (04.9)
Desired birth interval	
<2 years	44 (10.2)
2-3 years	218 (50.5)
>3 years	170 (39.4)

**Figure 1:**
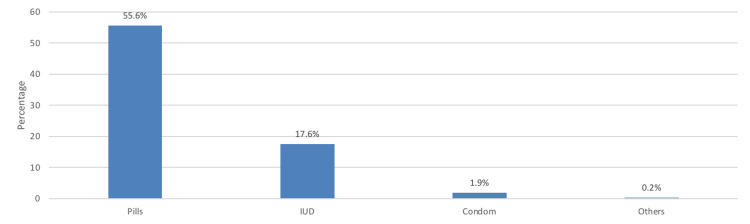
Types of modern contraceptive methods used by the study participants IUD: intrauterine device

The most commonly used contraceptive method was contraceptive pills (55.6%) followed by intrauterine devices (IUDs) (17.6%) (Figure 1).

**Figure 2:**
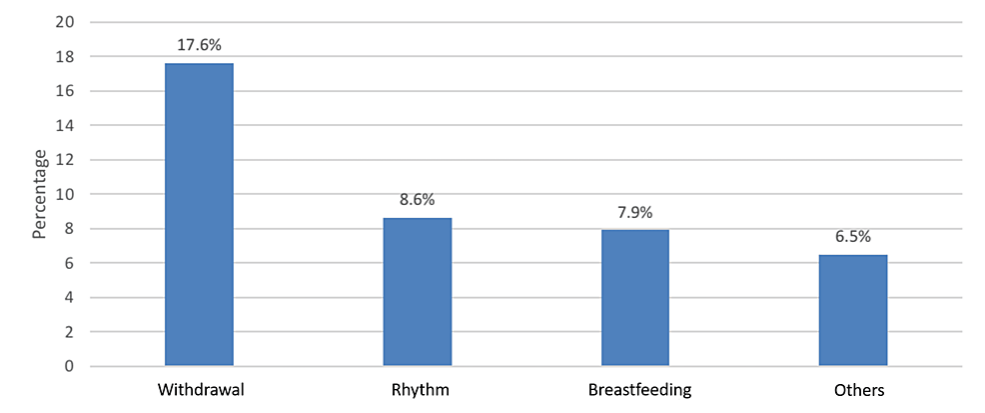
Types of conventional contraceptive methods used by the study population

The most commonly used conventional contraception methods were withdrawal (17.6%) and rhythm (8.6%) (Figure 2).

**Figure 3:**
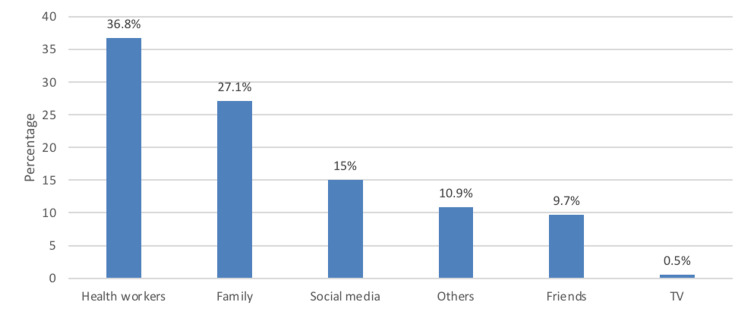
Source of information

The most frequently mentioned source of information was health workers (36.8%), followed by family (27.1%), and social media (15%) (Figure 3).

The percentage of women who were contraceptive users was 57.6%. Among those who were using contraceptives, the most common reason was the care of health (42.2%). Of the non-users, the most common reason was that it could cause health problems (41%) (Table 2).

**Table 2:** Use of contraception and reasons for acceptance and refusal (n=432)

Variables	N (%)
Use of contraceptives	
User	249 (57.6)
Non-user	183 (42.4)
Reason for contraceptive acceptance ^(n=249) ^	
Maternal	99 (39.8)
Care of health	105 (42.2)
Work/study	60 (24.1)
Others	38 (15.3)
Reason for refusal ^(n=183)^	
No reason	81 (44.3)
Children are a blessing	14 (07.7)
Cause of health problem	75 (41.0)
Affect mental health	04 (02.2)
Others	09 (04.9)

When measuring the relationship between the use of contraceptive methods and the sociodemographic characteristics of the women, it was observed that increasing intention to use contraceptive methods was associated with increasing family monthly income (p=0.035) and increasing parity (p<0.001). Also, the prevalence of contraceptive users was 97.2% of our population (Table 3).

**Table 3:** Relationship between the use of contraceptives and the sociodemographic characteristics of the study participants (n = 432)

Factor	Use of contraceptive method		p-value ^§^
	User N (%) ^(n=249)^	Non-user N (%) ^(n=183)^	
Age group			
<30 years	63 (25.3)	60 (32.8)	0.088
≥30 years	186 (74.7)	123 (67.2)	
Educational level			
High school or below	96 (38.6)	82 (44.8)	0.192
University education	153 (61.4)	101 (55.2)	
Family monthly income (SAR)			
<5,000	52 (20.9)	56 (30.6)	0.035 **
5,000 - 10,000	114 (45.8)	82 (44.8)	
>10,000	83 (33.3)	45 (24.6)	
Occupational status			
Unemployed	155 (62.2)	126 (68.9)	0.155
Employed/ Free business	94 (37.8)	57 (31.1)	
Parity			
None	12 (04.8)	41 (22.4)	<0.001 **
1 - 3	121 (48.6)	74 (40.4)	
>3	116 (46.6)	68 (37.2)	
Acceptance of using contraceptives			
Yes	242 (97.2)	123 (67.2)	<0.001 **
No	07 (02.8)	60 (32.8)	
Husband’s acceptance of using contraceptives			
Yes	231 (92.8)	118 (64.5)	<0.001 **
No	18 (07.2)	65 (35.5)	
Desired number of children			
3 or less	59 (23.7)	61 (33.3)	0.064
4-6	152 (61.0)	93 (50.8)	
7 or more	38 (15.3)	29 (15.8)	
Desired birth interval			
≤3 years	143 (57.4)	119 (65.0)	0.110
>3 years	106 (42.6)	64 (35.0)	
Source of information			
Family	67 (26.9)	50 (27.3)	0.055
Friends	26 (10.4)	16 (08.7)	
Social media	37 (14.9)	28 (15.3)	
Health workers	100 (40.2)	59 (32.2)	
Others	19 (07.6)	30 (16.4)	

A multivariate regression model revealed that women with parity of one to three, women who accepted contraceptive methods, and husbands who accepted contraceptive methods were the significant independent predictors of contraceptive use. This further suggests that compared to women without parity, women with a parity of one to three were predicted to increase the chance of using contraceptive methods by at least 5.1 times (adjusted odds ratio (AOR) = 5.104; 95% CI=2.357 - 11.051; p<0.001). Women who accepted contraceptive methods were 9.24 times more likely to use a contraceptive method (AOR=9.241; 95% CI=3.868-22.080; p<0.001). Also, women with husbands who accepted their use of contraceptives were 2.99-fold more likely to use contraceptive methods (AOR = 2.989; 95% CI=1.541-5.798; p=0.001). On the other hand, family monthly income did not show a significant effect of the use of contraceptive methods after adjustment to a regression model (Table 4).

**Table 4:** Multivariate regression analysis to determine the significant independent predictor associated with the use of contraceptives (n=432) AOR: adjusted odds ratio

Factor	AOR	95% CI	p-value
Family monthly income (SAR)			
<5,000	Ref		
5,000 - 10,000	1.642	0.898 – 3.002	0.107
>10,000	1.309	0.772 – 2.219	0.317
Parity			
None	Ref		
1 - 3	5.104	2.357 – 11.051	<0.001 **
>3	1.163	0.724 – 1.869	0.533
Acceptance of using contraceptives			
Yes	9.241	3.868 – 22.080	<0.001 **
No	Ref		
Husband’s acceptance of using contraceptives			
Yes	2.989	1.541 – 5.798	0.001 **
No	Ref		

## Discussion

The use of contraception has been growing in the past decades worldwide [15]. However, differences in the total use and the types of methods used were noted [16]. Among the 432 women included in our study, 57.6% were current users of different methods of contraception. Similarly, in a study by Jabbar et al. [17], 56% were using or had used some form of contraceptive. Our results identified better knowledge than previous studies, as 72.4% indicated their source of information to be healthcare workers. This is significantly higher than the study by Alsheeha et al. [6], who reported the main source of knowledge to be family members. However, the contraceptive method was similar to other Saudi studies, which reported contraceptive pills to be the most commonly used method of contraception [6,17,18]. 

The acceptance rate was high among both women and their husbands. The desired birthing interval was three years or less for the majority of participants. These results are similar to the majority of studies from Saudi Arabia, which could be attributed to Islamic teachings regarding the birth rate [5, 6].

The finding that parity was a significant predictor of contraceptive use is consistent with previous studies [6, 19, 20]. This is likely because women with more children are more likely to be concerned about spacing their births and preventing unintended pregnancies. The finding that acceptance of contraceptive methods was a significant predictor of contraceptive use is also consistent with previous studies [6, 19]. This is likely because women who accept contraceptive methods are more likely to be motivated to use them and to overcome any barriers to their use. The finding that the husband's acceptance was a significant predictor of contraceptive use is also consistent with previous studies [19]. This is likely because husbands can play a key role in supporting their wives' use of contraception.

The finding that family monthly income was not a significant predictor of contraceptive use after adjustment to a regression model is somewhat surprising. This is because previous studies have shown that financial resources can be a barrier to contraceptive use [6, 19]. This finding could possibly be due to contraceptives being available free of charge to the Saudi Arabian population, as well as a variety of contraceptive health services. However, the rates of contraceptive use among the studied participants were lower than the global rate of 62% and the rate in developed countries of 75% [21].

The study emphasized that despite being a developing and somewhat enclosed community, the prevalence of current and past contraceptive use is high and accepted in the area of the study. The study suggests that with increased efforts to raise awareness among the population, the prevalence of contraceptive use may reach the national standard. The findings of this study may be useful for healthcare providers and policymakers to develop effective strategies and interventions to increase the use of contraceptives and improve reproductive health outcomes among Saudi women as contraceptive use aids in preventing pregnancy-associated health risks and deaths in females, especially in teen pregnancies who have increased health risks due to early childbearing, and prevents pregnancies in older females who are also at increased risk of pregnancy-related health risks [22]. In addition to taking into consideration interbirth intervals, children born with a two-year interval of older siblings have a 60% increased risk of infant death; there is a 10% increase in risk for those with two to three intervals compared to the three-year interval [2]. Outside of contraceptive use, contraceptives are used in disorders such as polycystic ovary syndrome, dysmenorrhea, and endometriosis [23]. Additionally, the study highlights the importance of educating women and their husbands about the benefits of contraceptive use, as well as the importance of healthcare providers in providing accurate and reliable information on contraceptive methods.

The present study had some limitations. First, the study was conducted in an urban area in Saudi Arabia, so the results may not be generalizable to other areas of the country. Second, it is a cross-sectional study with more liability to interview and recall biases. Third, the sample size was relatively small.

## Conclusions

This study investigated the factors associated with contraceptive use among Saudi women. The results showed that women with one to three children, women who accepted contraceptive methods, and husbands who accepted contraceptive methods were the significant independent predictors of contraceptive use. This study provides new insights into the factors associated with contraceptive use among Saudi women. These findings can be used to develop interventions to increase contraceptive use among this population to improve outcomes related to women's health and interbirth intervals.
